# Gyroscope Pivot Bearing Dimension and Surface Defect Detection

**DOI:** 10.3390/s110303227

**Published:** 2011-03-16

**Authors:** Wenqian Ge, Huijie Zhao, Xudong Li

**Affiliations:** Precision Opto-Mechatronics Technology, Key-Laboratory of Education Ministry, Beijing University of Aeronautics and Astronautics, Beijing 100191, China; E-Mails: hjzhao@buaa.edu.cn (H.Z.); xdli@buaa.edu.cn (X.L.)

**Keywords:** illumination system, defect detection, pulse coupled neural network, particle swarm optimization, image segmentation

## Abstract

Because of the perceived lack of systematic analysis in illumination system design processes and a lack of criteria for design methods in vision detection a method for the design of a task-oriented illumination system is proposed. After detecting the micro-defects of a gyroscope pivot bearing with a high curvature glabrous surface and analyzing the characteristics of the surface detection and reflection model, a complex illumination system with coaxial and ring lights is proposed. The illumination system is then optimized based on the analysis of illuminance uniformity of target regions by simulation and grey scale uniformity and articulation that are calculated from grey imagery. Currently, in order to apply the Pulse Coupled Neural Network (PCNN) method, structural parameters must be tested and adjusted repeatedly. Therefore, this paper proposes the use of a particle swarm optimization (PSO) algorithm, in which the maximum between cluster variance rules is used as fitness function with a linearily reduced inertia factor. This algorithm is used to adaptively set PCNN connection coefficients and dynamic threshold, which avoids algorithmic precocity and local oscillations. The proposed method is used for pivot bearing defect image processing. The segmentation results of the maximum entropy and minimum error method and the one described in this paper are compared using buffer region matching, and the experimental results show that the method of this paper is effective.

## 1. Introduction

The gyroscope is the key part of an inertial navigation system, and its performance has a direct impact on the precision of the whole system. The gyroscope pivot bearing, whose diameter is only a few millimeters and its tolerance requirements are very strict, is a key part of a floating gyroscope, Its dimensions cannot be measured by contact methods after surface polishing. Any defects on the high curvature surface of the pivot bearing ball head are only a few microns in size and their shapes vary. As a result, it is difficult to measure dimensions and detect surface defects.

At present, new detection techniques such as image-based detection techniques, have become an indispensable feature in modern industrial production. During mass industrial production operations, image-based detection ensures the consistency of product detection and helps implement data quality monitoring and process control, thereby increasing detection security, reliability, efficiency and precision, and reducing production costs. According to the different characteristics detected image-based detection applications are categorized as dimension measurement, surface quality detection, structural quality detection and system operating status monitoring [1]. Among these applications, dimension measurement and surface quality detection are the most commonly used. Dimension measurement mainly involves characteristics of a target such as appearance, shape and position. It is also used in other fields, such as detection of discontinuous arc roundness in the field of machining [2], assemblage clearance in the field of automotive industry [3], and excursion and deflection of chips in the field of electronics, such as in printed circuit board (PCB) production [4]. Surface detection mainly involves detection of defects that impact product surface quality, such as fovea, scratches, cracks, air bladders, holes, wear, roughness, texture, and burrs, such as in steel plate surface defect detection [5], surface roughness measurement [6], tunnel wall surface defect detection [7], welding seam defect detection [8], fabric surface defect detection [9], and wood defect detection [10].

In this paper an image-based detection technique used for pivot bearing dimension measurement and surface defect detection is described. Illumination system design has a direct relationship with final imaging quality, and it is one of the keys for the success of any vision detection system. Improper illumination may give rise to many problems; for example, overexposure may hide true defects, shadows may cause edge false drops, and non-uniform illumination may cause image segmentation difficulties. As a result, illumination quality can directly impact image analysis results [11]. There are multiple methods for illumination system design; as an example of an optical path-based analysis, Lu [12] determined three color ring LED light source ray angles and light source geometric parameters, which were used for detecting circuit board weld defects; as a method based on optimization techniques, Sunil [13] studied the energy function of an optimum light source position, and calculated the minimum energy function and estimated light source position by a simulated annealing algorithm; for design based on ray directions, Nicolas [14] designed a light source with the same shape as a shower head, whi**c**h enhances defect contrast and detects parts surface defects with random direction scratches; as an example of design based on dynamic illumination, Ng [15] designed a moving ring light source and judged surface defects by bright ring changes on a slippery surface for the purpose of defect detection of bearing surfaces, and in an example of designs based on imaging quality analysis, Wu [16] associated image quality with measurement precision, and adjusted light illuminance by analyzing image quality to get highest measurement precision. In a word, one needs to study the light source optimization choice and design method combination for different detection tasks and work environments.

Moreover, the small proportions of the target defects relative to the entire picture in a micrograph, uneven surface illumination for high curvature surfaces and natural metal textures all contribute to make contrast of defect regions and background regions small, and image segmentation comparatively difficult. PCNN has been widely used in every field of image processing, such as denoising [17], segmentation [18], fusion [19] and feature extraction [20], but the elementary PCNN model framework is complex, and there are multiple undetermined parameters such as attenuation constants, amplification coefficients and connect coefficients. Most parameters are configured by artificial tests, which affect PCNN image processing speed and make it difficult to implement automatic image processing. Richard [21] used a genetic algorithm for setting optimum PCNN parameters, yet the key of genetic algorithms is the accurate setting of parameters such as variation and cross operator, which, if not set properly, will destroy the developmental stability. Particle Swarm Optimization (PSO) is an efficient search strategy [22], which features quick convergence and requires less parameter settings. Chao [23] used a PSO to search for the best parameter value of a generalized diffusion coefficient function that was used for anisotropic diffusion defect detection in low-contrast surface images. The PSO algorithm is used to automatically set PCNN optimization key parameters by fitness function of maxima between cluster variances, which carries out automatic PCNN image processing.

The paper is organized as follows: Section 2 introduces the structure of the gyroscope pivot bearing dimension measurement and surface detection system; Section 3 presents task-oriented illumination system design methods; Section 4 presents self-adaptive parameter settings obtained by integrating the PSO algorithm and PCNN; Section 5 describes experimental results and comparisons. Finally, some conclusions and future development are illustrated in Section 6.

## 2. Detection System Design

### 2.1. System Framework

**Figure 1.**
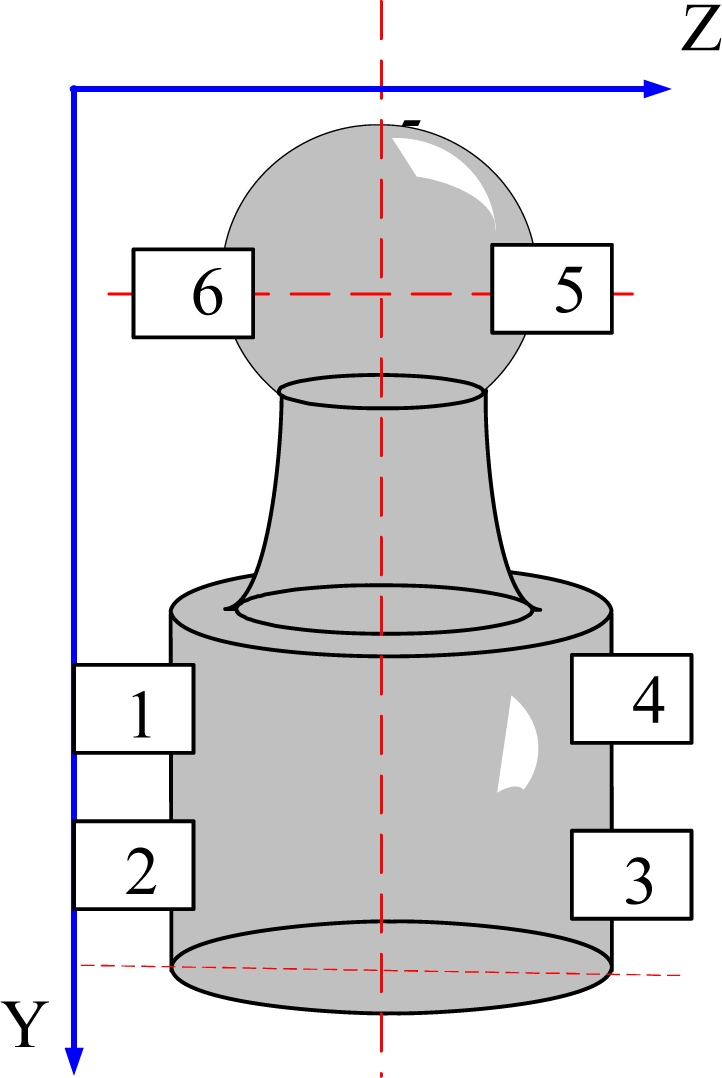
Drawing of a gyroscope pivot bearing.

**Figure 2.**
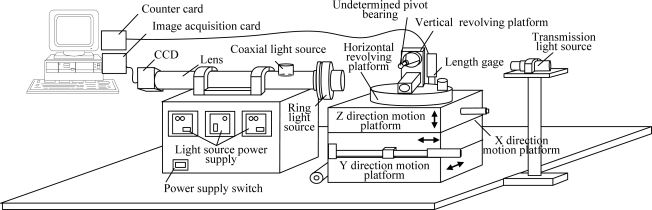
Gyroscope pivot bearing vision detection system.

The shape of a gyroscope pivot bearing is shown in Figure 1. The dimensions of gyroscope pivot bearings are small and any defects of its high curvature surface are at a micron-level; therefore some sort of amplificatory vision system is required, as shown in Figure 2, where the microscope is set horizontally, with coaxial and ring light sources which comprise a combination illumination system. The undetermined detection pivot bearing is installed on the combination motion platform that is composed of a three-dimension motion platform and a two-dimension revolving platform, where the Y direction and Z direction platform motion will implement pivot bearing position adjustment, which ensures that the undetermined target will be in the center of the visual field of the camera. The distance between the pivot bearing and lens is adjusted by the X direction motion platform, and this makes the lens focus clear. The two-dimension revolving platform providing horizontal and vertical circumrotation ensures the local surface ordinal will be in the visual field. Consequently it can implement the detection of all undetermined regions.

### 2.2. Pivot Bearing Surface Detection Policy

**Figure 3.**
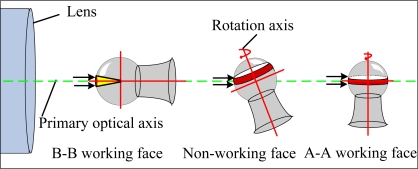
Detection sketch map for different working faces of pivot bearing surface.

As shown in Figure 3, above the hemisphere of the pivot bearing there is an undetermined detection region, the B-B working face, that is detected when the rotation axis and primary optical axis overlap. The vertical revolving platform is turned 360° to detect a zone on the non-working face, when the horizontal revolving platform is turned and pivot bearing has a certain inclination angle. The vertical revolving platform is turned 360° to detect the the A-A working face, when rotation axis and primary optical axis are upright, so that the top hemisphere can be detected.

### 2.3. Dimension Measurements

**Figure 4.**
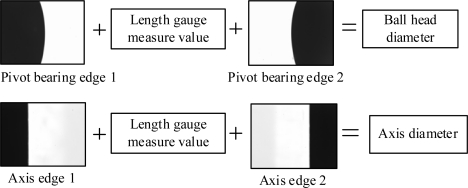
Sketch map of dimensions measurement.

The whole pivot bearing figure cannot be observed in one vision field when amplified, that is, only a partial edge is present in the vision field. Therefore, for dimension measurement it is necessary to move the pivot bearing and shoot images at multiple edge positions. Edge point coordinates are obtained by image processing, and the value of the length gauge is recorded when the Z direction motion platform is moved. The two edge section distances are calculated based on the edge point and recorded length gauge value. Figure 4 shows the undetermined geometry parameters. As shown in Figure 1, axis diameter is measured by shooting images at positions 1, 2, 3 and 4; ball head diameter is measured by shooting images at positions 5 and 6.

## 3. Illumination System Design

### 3.1. Task-Orientated Illumination Design Method

**Figure 5.**
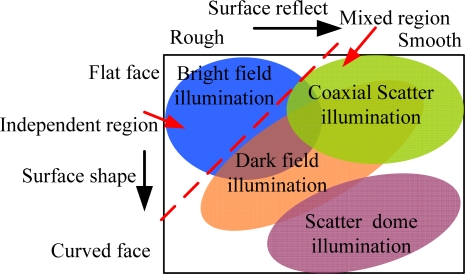
Relationship of light source selection and detected surface.

The vision detection illumination system design method in this paper can be summed up as follows:

1. Design of the illumination mode: First, the spatial running environment of the system is analyzed, including the effective visual field of the lens and the distance between the lens and the illumination surface, which preliminarily determines the basic structure of the illumination system. Second, undetermined illumination surface characteristics are analyzed. Different illumination modes are used based on the shapes and surface characteristics, as illustrated in Figure 5. The relatively flat and crude surface at the top left corner does not need any special illumination mode, but the geometric structure and distribution of light sources will become complex when the undetermined detection surface becomes slick bend along with rightward and downward movement.
2. Optic simulation assistance design: Uniformity is an important characteristic of light sources. Symmetrical illumination can give symmetrical gray scale images. Undetermined surface illuminance distribution may be impacted by light source structure, illumination distance and ray angles. Illumination system modeling is achieved using illumination optic theories and optic simulation software. Illuminated surface distribution is analyzed based on non-serial ray tracing of the model and the illumination effects of different illumination modes is simulated and compared.
3. Experimental research: Prepare enough testing samples, including undamaged samples, defective samples and exceptional samples; prepare standby testing light sources of different types and colors; use different types of light sources to beam on different positions of the target and observe the illumination effects.
4. Image analysis: Image quality is analyzed according to the detection task, which helps to optimize illumination system design. Selecting appropriate image evaluation methods and guidelines is very important. The estimate function may be used to evaluate image quality and guide illuminance design according to airspace or frequency characteristics of the images.

### 3.2. Gyroscope Pivot Bearing Illumination System Design

#### 3.2.1. Pivot Bearing Vision Detection System

Transmission light illumination is used to measure the pivot bearing dimensions. As shown in Figure 2, a transmission light source with a condenser lens in front can enhance ray parallelism, and one can then obtain high contrast edge images. Surface detection illumination is comparatively complex, and it is the primary research content in this paper.

**Figure 6.**
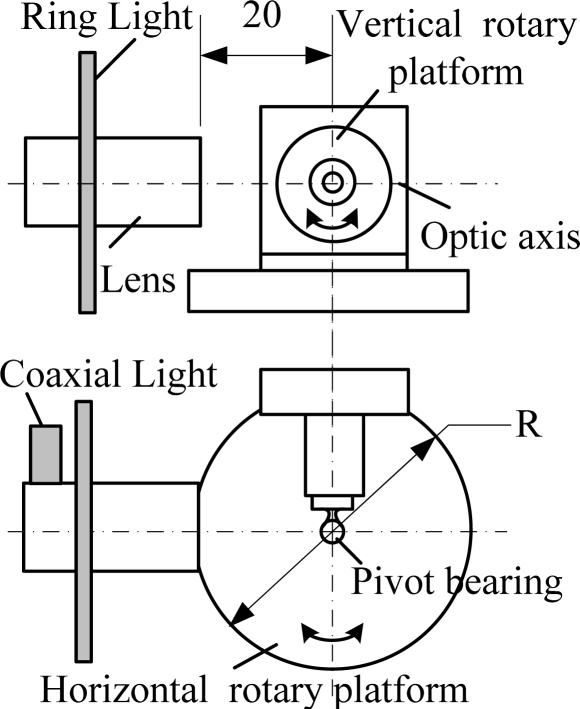
Framework map of the detection system.

The detection system used in the paper contains a Zoom 6000 series lens and a Mitutoyo amplification lens; objects can be amplified 25 times and the system depth of field is only 4 μm. Each effective visual field is limited and only the central region can be clearly imaged. The size of this region is about 0.085 mm × 0.085 mm, and the corresponding region in the image is about 240 × 240 pixels. Thereinafter this region will be called the target region. This paper designs a mechanical device composed of vertical and horizontal rotatory platforms (as shown in Figure 6), which can observe each region of ball surface by rotating the two platforms.

#### 3.2.2. Reflection Model Analysis

When rays reach an object there are three effects, reflection, transmission and absorption. Some geometric structure defects such as depressions, scratches and cracks can change the surface reflection. Surface property defects such as rust stains and blots may also cause changes in surface reflection and absorption. Any tiny structural region with defects induces regional roughness, which changes regional reflection characteristics.

**Figure 7.**
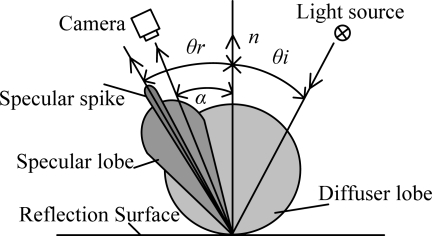
Reflection model.

Nayar compared the Beckmann-Spizzochino physical optics models with the Torrance-Sparrow geometrical optics model and proposed a unified reflectance framework for smooth and rough surfaces [24]. As shown in Figure 7, *θ_i_* is the angle of the incident rays, *α* is the direction of the camera, and *θ_r_* is main specula direction. Reflectance rays near the lens reflectance direction include the specular lobe *I_sl_*, specular spike *I_ss_* and diffuse lobe *I_dl_*, as shown in Equation (1):

(1) $$I_{\mathrm{im}}=I_{\mathrm{dl}}+I_{\mathrm{sl}}+I_{\mathrm{ss}}$$

The diffuse reflection component is represented by the Lambertian model, as shown in Equation (2) where *K_dl_* denotes the strength of the diffuse lobe and *θ_i_* is the angle of the incident rays:

(2) $$I_{\mathrm{dl}}=K_{\mathrm{dl}}\;\text{cos}\;\;\theta_{i}$$

Specular reflection can be denoted by the Torrance-Sparrow model due to its simpler mathematical form, as shown in Equation (3), where *K_sl_* is the magnitude of the specular lobe, and *D_k_* is the brae distributing function; *F* is the Fresnel coefficient; the geometric attenuation factor *G* describes the shadowing and masking effects of facets by adjacent facets:

(3) $$I_{\mathrm{sl}}=K_{\mathrm{sl}}D_{k}\mathrm{FG}$$

The specular spike component is a very sharp function which is approximated by the delta function, as shown in Equation (4), where *K_ss_* is the strength of the specular spike component:

(4) $$I_{\mathrm{ss}}=K_{\mathrm{ss}}\delta (\theta_{i}-\theta_{r})\delta (\phi_{r})$$

The main constituent of the surface reflection is judged by the relationship between the high standard deviation *σ_h_* of the illuminated surface and the incident wavelength *λ*, as shown in (5). Franz [25] considered that the specular spike effect can be ignored when *E* is greater than 1.5. Therefore, the specular reflection is the major component for rough surfaces, and the specular spike can be ignored; for smooth or defective surfaces, the specular spike is the main factor considered:

(5) $$E=\frac{\sigma_{h}}{\lambda }$$

The surface roughness class is 13 (*R_a_* < 0.025 *μm*) after a pivot bearing surface is polished; then:

(6) $$\frac{0.025\times 10^{-6}}{555\times 10^{-9}}=0.045<1.5$$

Therefore, this provides a specular spike model for pivot bearing regions with no defects, but roughness increases for a region with defects where specular reflection applies.

#### 3.2.3. Pivot Bearing Illumination Mode Research

**Figure 8.**
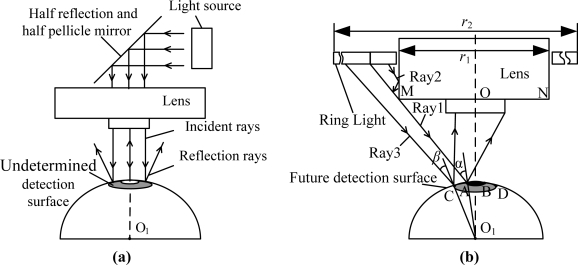
Light source design. **(a)** Coaxial light, **(b)** Ring light source.

Rays pass half reflection and half pellicle mirrors in the lens, where coaxial light is sent up from and reaches the illuminated surface, as shown in Figure 8(a). Rays from the lens center reach the illuminated surface vertically, and the rays can be reflected to the lens, but non-primary optic axis incident rays cannot be reflected to the lens as incidence angles becoming bigger because of the curved illuminated surface. As a result, the brightness of the illuminated surface center is higher and decreases gradually on the surrounding surface when only coaxial light is used.

A ring light is installed on the lense when it is used as a light source, as shown in Figure 8(b), and there is a hole with diameter *r*_1_ in the light source center. Ray 1 beams on point A of the illuminated surface and the angle of incidence is *α*, which comes into the entrance pupil of the lens by surface reflection. The incidence angle is greater than *α* when rays except ray 1 beam on the arc AB, and the rays cannot come into the entrance pupil of lens by surface reflection. The rays (like ray 2) within ray 1 have smaller incidence angles for arc AB, but these rays cannot reach the illuminated surface because they are sheltered by segment MN of the lens. Although incident rays can reach arc AB, the angle of incidence is smaller than the critical angle *α*. As a result, no rays can come into the lens, that is, the ball crown corresponding to arc AB is always dark. When the rays (like ray 3) except ray 1 reach arc AB, some rays can come into entrance pupil of the lens when the incidence angle changes. Moreover incidence angle *β* decreases gradually along with point C being apart from the main coaxial O_1_O. Consequently, the reflected rays that come into lens increase gradually when the rays are apart from the main coaxial. Coaxial light and ring light form a complex illumination system, as shown in Figure 6, which balances the target region illumination.

### 3.3. Emulation and Experimental Research

#### 3.3.1. Illuminance Uniformity Emulation

**Figure 9.**
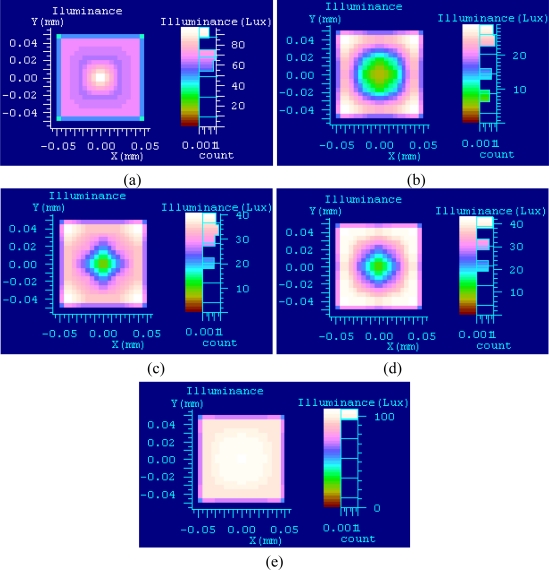
Illumination diagram: **(a)** coaxial light only; **(b)**, **(c)**, **(d)** ring light only, L is 35 mm, 40 mm, 45 mm in turn; **(e)** complex illumination.

The LightTools software can be used for computer-aided design of the illumination system by illumination optics. LightTools was used to emulate and analyze surface illuminance uniformity by using the coaxial and ring light that form the complex illumination system described in this paper. The illuminated object is a ball whose diameter is 0.5 mm when the system model is built, the distance of the emergent surface of coaxial light and illumination surface is 20 mm, and the illuminated surface has specular reflection. The diameter of the ring light is equal to the lens inside diameter *r*_1_ = 30 mm, and the outside diameter is 90 mm. Twenty thousand rays are used to trace when only coaxial light is used. The illuminance diagram is shown in Figure 9(a), where the view on the left is a two dimensional grating diagram, with x-coordinates and y-coordinates denoting object size. The view on the right is a histogram, where different colors denote different illuminance classes. Illuminance of target center is higher as shown in the figure, and the further from the center the lower the illuminance.

One hundred thousand rays are used to trace when only the ring light is used. The distance of the ring light and illuminated surface is limited at 35 to 45 mm because of space limitations of the vision system. Assume that the distances are 35 mm, 40 mm and 45 mm. The illuminance diagram is shown in Figure 9(b–d). The illuminance of the target center is low; therefore, the further from the center the higher the illuminance. The illuminance diagram is shown in Figure 9(e), when complex illumination is used. Only the illuminance diagram with the distance being 35 mm is illustrated because the effect of others is similar. Illuminance is even in the target region.

Illuminance uniformity is evaluated by standard criteria of regional illuminance, and the standard criteria of a target region are listed in Table 1. The distance of ring light and illuminated surface is L using complex illumination. Illuminance uniformity is better when complex illumination is used, as standard criteria decrease and L decreases.

#### 3.3.2. Experiment and Grey Scale Image Analysis

**Figure 10.**
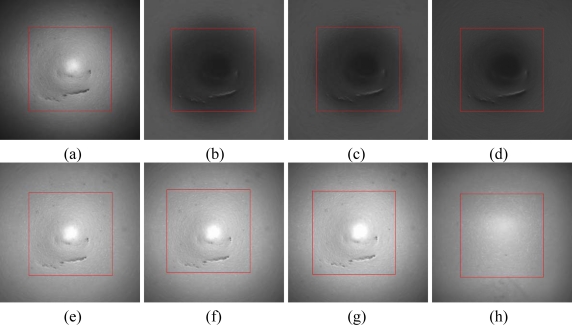
Gray scale image of target region: **(a)** coaxial light only, **(b)**, **(c)**, **(d)** ring light only, L is 35 mm, 40 mm, 45mm in turn, **(e)**, **(f)**, **(g)** combining light, L is 35 mm, 40 mm, 45 mm in turn, **(h)** no defect.

If direct light is used to illuminate the surface directly the camera is easily saturated due to specular reflection. Moreover, the reflexion will change because of the tiny angle changes of the light source, illuminated surface and lens. In this paper the ring light source uses scatter illumination. Grey-scale images are shown in Figure 10, with the target region framed. The bright spot in Figure 10(a,e–g) is because of the strong regional reflection caused by the irregular ball surface after machining, the gray scale of Figure 10(h) is even greater because the surface has no defects. Coaxial light and ring light illuminance are both bright field illumination. Reflected rays from defects, for example for depressions or scratches, cannot come into the lens. As a result, there is a low gray scale region that contrasts with the background. More analysis of the gray-scale images of the target region follows.

1. Target region gray scale uniformity analysis: Illuminance uniformity is estimated by gray scale uniformity U of the target region, where a lower value indicates better uniformity. As shown in Equation (7), variance and mean gray scale are denoted by *Var* and *Ave*, respectively. The computed results of Figure 10(a,e–g) are listed in Table 2, uniformity using complex illumination is higher than that for coaxial light, and it becomes better when L increases:
  (7) $$U=\frac{\mathrm{Var}}{\mathrm{Ave}}$$
  (8) $$f(I)=\sum_{x}\sum_{y}{\left[I(x+z,\;\;y)-I(x,\;\;y)\right]}^{2}$$
2. Image articulation analysis: Blurring appears to a certain extent around the target region when complex illuminance is used. In addition, the articulation of the target region will change along with the change of ring light distance L. In this paper the articulation of the target region is calculated by the Brenner function [26], which is calculated by differences of neighboring pixel gray scales, square and sum. The greater the value is, the higher the articulation is, as shown in Equation (8), where *l* denotes gray scale (after normalization), and *z* denotes pixel interval and is usually 1. Blurring will present around the target region boundary, thereby articulation is calculated in the boundary region. The region is calculated between 200 × 200 to 240 × 240 pixels from the image center. The results of Figure 10(a,e–g) are listed in Table. When L is 35 mm, the Brenner function has the greatest value, and the articulation is the best.

## 4. PSO-PCNN Image Processing

### 4.1. PCNN Mathematical Model

**Figure 11.**
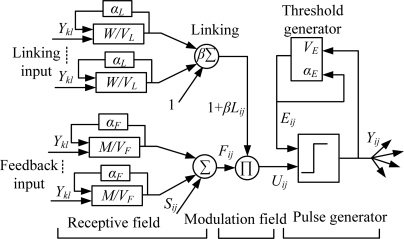
PCNN neural structure drawing.

As shown in Figure 11, each neuron contains the input field, feedback field and pulse generator field [27]. The feedback field can receive exterior and local stimulation, and the input field can receive only local stimulation. Every neuron is connect to a neighboring field by the corresponding weight matrix and features attenuation delay, as shown in Equations (9) and (10):

(9) $$F_{\mathrm{ij}}\;(n)=e^{-\alpha_{F}}F_{\mathrm{ij}}\;(n-1)+S_{\mathrm{ij}}+V_{F}\sum_{\mathrm{kl}}M_{\mathrm{ijkl}}Y_{\mathrm{kl}}\;(n-1)$$

(10) $$L_{\mathrm{ij}}\;(n)=e^{-\alpha_{L}}L_{\mathrm{ij}}\;(n-1)+V_{L}\sum_{\mathrm{kl}}W_{\mathrm{ijkl}}Y_{\mathrm{kl}}\;(n-1)$$

where *F*_ij_ is the feedback input of neuron *N*_ij_ in the two-dimension neural network, and *L*_ij_ is linking item, which remembers former states and has the exponential attenuation form. *Y*_kl_ is the neuron output of iteration (n − 1), *V_F_* and *V_L_* are amplification coefficients of the feedback field and linking field, respectively; *α_F_* and *α_L_* are attenuation time constants of the feedback field and linking field respectively. Internal activity items are generated by non-linearity coupling modulation of feedback input using the linking field, as shown in Equation (11), the value of which determines if a neuron generates pulses. Modulation intensity is decided by linking coefficient *β*:

(11) $$U_{\mathrm{ij}}\;(n)=F_{\mathrm{ij}}\;(n)[1+{\beta L}_{\mathrm{ij}}\;(n)]$$

Pulses will be generated if the internal activity items are greater than the dynamic threshold, as shown in Equation (12):

(12) $$Y_{\mathrm{ij}}=\{\begin{array}{ll} 1 & U_{\mathrm{ij}}\;(n)>E_{\mathrm{ij}}\;(n-1)\\ 0 & \text{other} \end{array}$$

The dynamic threshold is denoted by Equation (13), where *V_E_* and *α_E_* denote the amplification coefficient and attenuation time constant of the dynamic threshold, respectively:

(13) $$E_{\mathrm{ij}}\;(n)=e^{-\alpha_{E}}E_{\mathrm{ij}}\;(n-1)+V_{E}Y_{\mathrm{ij}}\;(n-1)$$

(14) $$U_{\mathrm{ij}}\;(n)=F_{\mathrm{ij}}\;(n)[1+{\beta L}_{\mathrm{ij}}\;(n)]$$

### 4.2. PCNN Model Simplification and its Image Processing

Neurons have a one-to-one relationship with image pixels, which constructs a single layered two-dimension and local connection network when PCNN is used for image processing. Shi [18] simplified the input field and recomposed the dynamic threshold to be a linearly decreased one with a constant that is calculated by derivation of the contrast (DOC). Lu [28] improved the region growing PCNN model by modifying the linking channel function and decreased the complexity of adjusting parameters.

In this paper, the basal model structure of PCNN will be simplified hereinafter. It is simplified at the input field: *F*_ij_(*n*) = *S*_ij_(*n*). The neighborhood action part is omitted in the feedback field, of which the answer to a neighbor field action is boiled down to linking coefficient action for the linking field. *L*_ij_(*n*) = *V_L_* ∑_kl_ *W_ijkl_Y*_kl_(*n* – 1). Attenuation items are omitted in both the feedback and linking fields, which reduces the number of structure parameters and decreases computational requirements for confirming undetermined parameters. Meanwhile, the basic characteristics of the PCNN model are retained. In this case, the internal activity item is denoted by Equation (15):

(15) $$U_{\mathrm{ij}}\;(n)=S_{\mathrm{ij}}\;(n)[1+{\beta L}_{\mathrm{ij}}\;(n)]$$

Neurons *N*_ij_ and *N*_kl_ are hypothetically linked, where exterior stimulations are *S*_ij_ and *S*_kl_ respectively and *S*_ij_ > *S*_kl_. Initially, when neurons are not connected to each other, the greater the input value the higher ignition frequency. That is, a high gray scale pixel will ignite first. The temporary dynamic threshold of the two neurons is 0, and internal activity items are greater than the dynamic threshold. The dynamic threshold increases to *V_E_* immediately after the first ignition and pulse export. At the same time, exported pulses of the two neurons come into the linking field of each other, which increases the internal state, but the neurons will not ignite immediately as *V_E_* was set to a high value. Neuron *N*_ij_ will first generate the second ignition because the exterior stimulation of neuron *N*_ij_ is greater than *N*_kl_. Meanwhile, neuron *N*_kl_ receives a pulse input from neuron *N*_ij_ through the linking field, which increases the internal state to *U*_kl_ = *S*_kl_(1 + *βL_kl_*) by couple modulation. If *U*_kl_ > *E*_kl_(n) at that time, neuron *N*_kl_ can ignite ahead of time, indicating that neuron *N*_kl_ is captured by neuron *N*_ij_. Then, the two neurons can synchronously ignite. This capture characteristic is applicable to a neuron and other neurons in its neighboring field. PCNN generates pulses as comparability swarm, which enables neurons with similar properties to synchronously ignite.

The linking weight matrix can be set to 4-connection, 8-connection or others according to actual requirements. The center pixel is affected by the distance of a pixel and its neighboring pixel, that is, if information transfer is strong from a neighboring field to the center. The nearer to the center pixel the greater the weight. *W_ijkl_* is calculated by Eyckid distance quadratic sum reciprocal of neighborhood neuron and current neuron, namely:

(16) $$W_{\mathrm{ijkl}}=\frac{1}{{\left(i-k\right)}^{2}+{\left(j-l\right)}^{2}}$$

At the same time, neuron *N*_ij_ generates pulses that can capture distant neurons by neuron transfer due to the pulse transmission characteristic of PCNN. Consequently, its segmentation result features better self-adaption than traditional threshold segmentation methods.

### 4.3. PSO-Based Parameter Self-Adaption

#### 4.3.1. PSO Algorithm

The global optimum is searched in parameter space by a PSO algorithm using some particles [29]. Each particle of the population denotes a potential solution. A global optimum will be achieved and an optimal value will be obtained by information exchange among particles and iterative evolution. Assume that the position and velocity of the *i*th particle are represented as *X_i_* = (*X*_*i*1_,*X*_*i*2_,⋯,*X_id_*) and *V_i_* = (*V*_*i*1_,*V*_*i*2_,⋯,*V_id_*), respectively, in *d*-dimension search space. Particles will renovate automatically by two optimum solutions upon iteration: one is the optimal position *P_i_* = (*P*_*i*1_,*P*_*i*2_,⋯,*P_id_*) that has been found by the particle itself; the other one is the optimal position *P_g_* = (*P*_*g*1_,*P*_*g*2_,⋯,*P_gd_*) that has been located among the whole population. Each particle renovates its velocity and position by Equations (17) and (18):

(17) $$v_{\mathrm{ij}}^{\left(t+1\right)}={\mathrm{wv}}_{\mathrm{ij}}^{\left(t\right)}+c_{1}r_{1}[p_{\mathrm{ij}}-x_{\mathrm{ij}}^{\left(t\right)}]+c_{2}r_{2}[p_{\mathrm{gj}}-x_{\mathrm{ij}}^{\left(t\right)}]$$

(18) $$x_{\mathrm{ij}}^{\left(t+1\right)}=x_{\mathrm{ij}}^{\left(t\right)}+v_{\mathrm{ij}}^{\left(t+1\right)}$$

where *c*_1_ and *c*_2_ are learning factors, *t* is the number of iterations, *r*_1_ and *r*_2_ are two random numbers within the range of 0 to 1, and *w* is an inertia factor. The linearity reduced inertia factor is used to avoid PSO algorithm precocity and oscillation near the global optimal solution, as shown in Equation (19):

(19) $$w=w_{\text{max}}-t\frac{w_{\text{max}}-w_{\text{min}}}{M_{\mathrm{num}}}$$

where *t* denotes the current iteration number, and *M_num_* denotes the total iteration number. The inertia factor decreases linearly from the maximum to the minimum. It is propitious to leave from the local minimal point and easy for global search when the inertia factor is great and propitious for accurate local search and algorithm convergence when the inertia factor is small.

#### 4.3.2. PSO-PCNN Parameters Configuration

The number of undetermined parameters for the simplified PCNN model has been greatly decreased, with only *β*, *W_ijkl_*, *V_L_*, *V_E_* and *α_E_* left. *V_L_*, *W_ijkl_* and *α_E_* have lower impact on segmentation results; *β* and *V_E_* have higher impact, and need to be set differently for different images. Therefore, the two key parameters are optimized by the PSO algorithm in the paper. The maximum cluster variance denotes a low probability of background misclassification. The maximum between cluster variance rules is used as the fitness function in this paper. It is defined as follows:

(20) $$\sigma^{2}=p_{0}{\left(\mu_{0}-\mu_{T}\right)}^{2}+p_{1}{\left(\mu_{1}-\mu_{T}\right)}^{2}$$

where $p_{0}=\sum_{p_{i}\in A}p_{i}$, $p_{1}=\sum_{p_{i}\in B}p_{i}=1-p_{0}$, *A* denotes the target region of binary image after PCNN segmentation, *B* denotes the corresponding background region, *p_i_* denotes the probability of each gray scale, and *μ*_0_, *μ*_1_, and *μ_T_* denote the mean gray scale of the target region, background region and image respectively.

The key parameters *β* and *V_E_* of PCNN are searched by the PSO algorithm with a linearity reduced inertia factor. The procedure is as follows:

1. Initialize the position and values of each particle in the population, ***P****_i_* = (*β*, *V_E_*);
2. Compute the fitness value of each particle. The particle position vector is imported to the PCNN model, and image segmentation is executed. The output binary image is mapped to the target of the original image and background region. Compute the variance between clusters as fitness value using Equation (20);
3. The position and fitness value of current particle are saved in the individual best position (*pBest*), and the position and fitness value of all the *pBest* are saved in the article population best position (*gBest*);
4. Update the inertia factor *w* using Equation(19);
5. Update the velocity $v_{\mathrm{ij}}^{\left(t+1\right)}$ and position $x_{\mathrm{ij}}^{\left(t+1\right)}$ of each particle using Equations (17) and (18);
6. If the fitness value of a particle is better than a value with the best historical position, set position *pBest* as the current position for the particle; if a fitness value of particle population is better than the best historical position among the population, set position *gBest* as the current position for the particle;
7. Assume that *t* = *t* + 1. Return to step (2), until *t* = *M_num_*;
8. The best solution position is *gBest* by iteration, and the optimizing *β* and *V_E_* are used for PCNN image segmentation.

## 5. Experiments

### 5.1. Experiment One: Dimension Measurement

1. Axis diameter measurement: Axis diameter measurement can be considered as a distance measurement between parallel lines, and the premise is fitting parallel lines by obtaining the two groups of edge data. Edge points after image processing are processed by least squares fitting to obtain two line equations, which makes the two line slopes equal. Then the distance of two lines will be calculated. Point numbers of the two lines are denoted by *n*_1_ and *n*_2_, point sets of the two lines are *P*_1_ and *P*_2_, and the line equations are:
  (21) $$\begin{array}{l} x\text{sin}\;\;\theta -y\text{cos}\;\;\theta +d_{1}=0\\ x\text{sin}\;\theta -y\;\text{cos}\;\;\theta +d_{2}=0 \end{array}$$
  The target function is constructed by least square fitting as shown in Equation (22):
  (22) $$\begin{array}{c} E\left(\theta ,\;d_{1},\;d_{2},\;\cdot \cdot \cdot ,\;d_{m}\right)=\sum_{\left(x_{i},\;y_{i}\right)\in P_{1}}{\left[x_{i}\;\text{sin}\;\theta -y_{i}\;\text{cos}\;\theta +d_{1}\right]}^{2}+\sum_{\left(x_{i},\;y_{i}\right)\in P_{2}}{\left[x_{i}\;\text{sin}\;\theta -y_{i}\;\text{cos}\;\theta +d_{2}\right]}^{2}\\ {\bar{x}}_{j}=\sum_{\left(x_{i},\;y_{i}\right)\in P_{j}}x_{i},\;{\bar{y}}_{j}=\sum_{\left(x_{i},\;y_{i}\right)\in P_{j}}y_{i},\;(j=1,\;2),\;\;\;\;(i=1,\;2,\;\cdots ,\;n_{j}),\; \end{array}$$
  then
  (23) $$\begin{array}{ll} a_{j}=\sum_{\left(x_{i},\;y_{i}\right)\in P_{j}}x_{i}^{2}-{\bar{x}}_{j}^{2}-\sum_{\left(x_{i},\;y_{i}\right)\in P_{j}}y_{i}^{2}+{\bar{y}}_{j}^{2} & \\ & j=1,\;2,\;\;,\;\;\;\;\;i=1,\;2,\;\cdots ,\;n_{j}\\ b_{j}=\sum_{\left(x_{i},\;y_{i}\right)\in P_{j}}x_{i}y_{i}-{\bar{x}}_{j}{\bar{y}}_{j} & \end{array}$$
  Assuming: $\alpha =\sum_{j-1}^{m}a_{j}$, $b=\sum_{j-1}^{m}b_{j}$, (*j* = 1,2), then *α* sin 2*θ* − 2*b* cos 2*θ* = 0, when *b*≠0, $\text{tan}\;\theta =\frac{-a+\sqrt{a^{2}+{4b}^{2}}}{2b}$, using *d_j_* = *ȳ_j_* cos *θ* − *x̄_j_* sin *θ*, (*j* = 1,2), obtain *d_j_*, and two parallel line equations. Then the distance of the two lines will be obtained, which is the axis diameter.
2. Ball head diameter measurement: Two arc segments cannot form a perfect circle due to the machining course of a ball. Therefore, the ball head diameter is calculated by averaging multiple results of the maximum distance of the left and right arc segments.
3. Experimental results: Ten pivot bearings are measured using the system for validating measurement methods. Compareison of the results with the results obtained by contact measurement using length measuring instruments (measurement accuracy is 0.0002 mm). Table 3 shows that the difference of measurement results is less than 0.001 mm.

### 5.2. Experiment 2: Defect Image Detection

**Figure 12.**
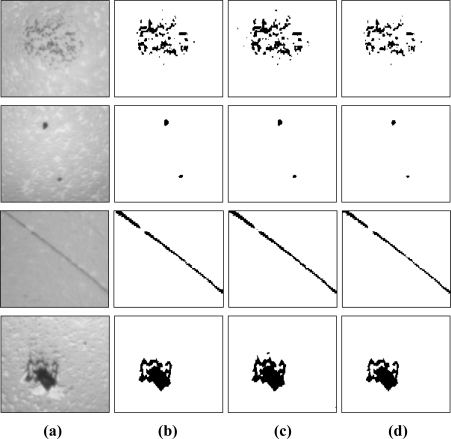
Result of image processing, **(a)** Original image with defect, **(b)** Result of this paper, **(c)** Result of maximum entropy, **(d)** Result of minimum error.

There are many reasons that may cause pivot bearing surface defects, for example non-uniform material structure, lapping stress and polishing, which all result in different defect dimensions and modes. Images of pivot bearing surface defects are segmented for validating the algorithm put forward in this paper. *V_L_* and *α_E_* in the PCNN model are 1, *W_ijkl_* is set by Equation (16), where the size of the neighbor field is 5 × 5. PSO inertia factors in the experiment are *w*_max_ = 0.9 and *w*_min_ = 0.4; the iteration number is *M_num_* = 20; the number of particle populations is *N* = 10; the learning factor is *c*_1_ = *c*_2_ = 2, as suggested by Shi [30]. Undetermined optimal PCNN parameters *β* and *V_E_* are initialized in the PSO algorithm, and the optimum solution will be used for the PCNN parameters by an iteration search. The experiments were conducted on an Intel Pentium 4 CPU 3.0 GHz personal computer. Processing time of the PCNN algorithm is about 3 s on average. Image processing results for various defect images using methods in this paper are compared with others as shown in Figure 12. It shows rust stains, macula, coarse threads and depressions from top down, and original image and processing results of this paper, maximum entropy and minimum errors from left to right.

**Figure 13.**
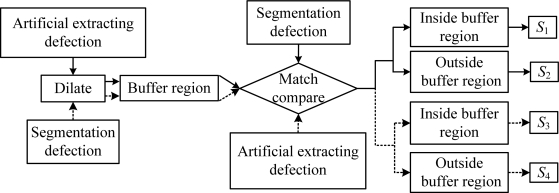
Flowchart of buffer region matching.

Sunil [31] used the buffer region matching method [32] to estimate segmentation results of concrete infrastructure crack images. We use for reference Sunil’s method to objectively estimate segmentation results in this paper. The algorithm flow chart is shown in Figure 13. Buffer regions are formed by 3 × 3 morphological dilate operation for defect regions that are extracted by artificial processing, and then defects that have been segmented are matched and compared with the buffer region. The pixels are denoted as *S*_1_ inside the buffer region and as *S*_2_ outside that region. Similarly, buffer regions are formed by dilate operation for defect targets that are extracted by segmentation methods, and those are compared with defects that are extracted by artificial processing. The pixels are denoted as *S*_3_ and *S*_4_ inside and outside the buffer region, respectively. Image segmentation are estimated by three estimating factors in Equations (24–26), where *C* denotes Correctness that is the correct degree of the defect target region as shown in Equation (24); *I* denotes Integrality that is the overlay degree of artificial distilling defects by segmentation processing as shown in (25); *Q* denotes Quality, which is a synthetic estimation of correctness and integrality as shown in Equation (26).

(24) $$C=\frac{S_{1}}{S_{1}+S_{2}}$$

(25) $$I=\frac{S_{3}}{S_{3}+S_{4}}$$

(26) $$Q=\frac{S_{1}}{S_{1}+S_{2}+S_{4}}$$

(27) $$S=k_{1}C+k_{2}I+k_{3}Q$$

The ideal value of the three estimation factors is 1. A value closer to 1 indicates higher performance. The estimation factor results of pivot bearing surface defect processing are listed Table 4, where the three estimation factors for coarse thread and macula processing results in this paper are better than the other two.

The correctness of rust stains processing results is not the best, but the integrality and quality are better. Integrality of fovea processing is not as good as maximum entropy, but the correctness and quality are better. The synthetic estimation factor is calculated by Equation (27) that is proposed in this paper to synthetically compare the three algorithm processing results for different defects, where *k*_1_, *k*_2_, *k*_3_ are the coefficients of the three estimation factors, and *k*_1_ = *k*_2_ = *k*_3_ = ⅓ in this paper. Results are shown in Table 5. Comparing the results for different defects, the PCNN processing results in this paper are better than the other two, and that proves the methods in this paper can be used for defect image segmentation.

## 6. Conclusions

An image analysis-based vision detection system aimed at gyroscope pivot bearing dimension measurement and surface defect detection is described in this paper. It implements pivot bearing axis diameter and ball head diameter measurement and surface defect detection in one instrument. Illumination has a direct effect on imaging quality and detection results. Therefore, stepwise design, simulation, experiment and analysis are used to propose an illumination system design method. Detection target characteristics and detection requirements are both considered, and the illumination model is designed according to the system environment. Illuminance uniformity is simulated and image results are analyzed by experimental research, which optimize illumination system design. Complex illumination is composed by a coaxial light and a ring light source with the purpose of gyroscope pivot bearing surface defect detection, which enhance illuminance uniformity and image articulation of target regions. Furthermore, the PSO-based PCNN method is proposed in this paper to process pivot bearing defect surfaces, and two key PCNN parameters, connect coefficient and dynamic threshold, are optimized by the PSO algorithm using the maximum cluster variance as a fitness function. Linearity reduced inertia factor is adopted to avoid PSO algorithmic precocity and oscillation near the global optimal solution, which implements a self-adaptive PCNN parameter setting. Buffer regions matching the estimated method for segmentation results prove that the methods in this paper can be used for image segmentation. In addition, iterative computation is required for both PSO and PCNN, therefore how to improve the speed of PSO algorithm-based optimal computation for PCNN parameters still needs further study.

## Figures and Tables

**Table 1.** Standard criteria for target region illuminance.

**Coaxial light**	**Combining illumination**		
	**L = 35 mm**	**L = 40 mm**	**L = 45 mm**
6.8	1.8	2.2	3.3

**Table 2.** Uniformity and articulation of image in different illuminance modes.

**Illuminance mode**		***U***	**Brenner**
Coaxial light		3.58	1.14
Combination light	L = 35	1.17	4.37
	L = 40	1.33	3.85
	L = 45	1.49	2.51

**Table 3.** Measurement results of axis diameter and ball head diameter (mm).

	Workpiece number	1	2	3	4	5	6	7	8	9	10
Axis diameter	System measurement	3.0015	3.0016	3.0015	3.0013	3.0011	3.0016	3.0006	3.0012	3.0001	3.0018
	Length measuring instruments	3.0018	3.0011	3.0019	3.0016	3.0008	3.0012	3.0004	3.0015	3.0005	3.0016
	Difference	−0.0003	0.0005	−0.0004	−0.0003	0.0003	0.0004	0.0002	−0.0003	−0.0004	0.0002
Ball head diameter	System measurement	0.4944	0.495	0.4968	0.4971	0.4967	0.4985	0.4978	0.4968	0.4936	0.4935
	Length measuring instruments	0.4941	0.4955	0.4965	0.4969	0.4965	0.4989	0.4985	0.497	0.4939	0.494
	Difference	0.0003	−0.0005	0.0003	0.0002	0.0002	−0.0004	−0.0002	−0.0002	−0.0003	−0.0005

**Table 4.** Result of buffer region matching estimate.

**Defect**	**Segmentation Method**	***C***	***I***	***Q***	**Defect**	**Segmentation Method**	***C***	***I***	***Q***
Rust stains	This paper	0.971	0.983	0.955	Macula	This paper	0.98	0.877	0.792
	Minimum error	0.979	0.841	0.8		Minimum error	0.966	0.714	0.597
	Maximum entropy	0.987	0.908	0.889		Maximum entropy	0.921	0.844	0.773
Coarse thread	This paper	0.98	0.867	0.792	Fovea	This paper	0.973	0.985	0.963
	Minimum error	0.931	0.844	0.773		Minimum error	0.979	0.948	0.927
	Maximum entropy	0.976	0.714	0.597		Maximum entropy	0.882	0.991	0.876

**Table 5.** *S* estimation factor results.

	**Rust stains**	**Macula**	**Coarse thread**	**Fovea**
This paper	0.9697	0.883	0.8797	0.9736
Minimum error	0.8733	0.759	0.8493	0.9513
Maximum entropy	0.928	0.846	0.7623	0.9163
